# Annual acknowledgement of manuscript reviewers

**DOI:** 10.1186/2047-9158-2-3

**Published:** 2013-03-19

**Authors:** Shengdi Chen

**Affiliations:** 1 Ruijin Hospital, Shanghai Jiaotong University School of Medicine, Shanghai, China

## Abstract

**Contributing reviewers:**

The editors of *Translational Neurodegeneration* would like to thank all our reviewers who have contributed to the journal in Volume 1 (2012).

## 

Clive Ballard

United Kingdom

Larry Baum

Hong Kong

Carsten Buhmann

Germany

Xiaochun Chen

China

Yaomin Chen

United States of America

Jianqing Ding

China

Xiang Gao

United States of America

Serge Gauthier

Canada

Howard Gendelman

United States of America

Glenda Halliday

Australia

Yingbo He

United States of America

Yue Huang

Australia

Jianping Jia

China

Ying Jin

China

Sheng-Han Kuo

United States of America

Debomoy Lahiri

United States of America

Rena Li

United States of America

Xiao-Jiang Li

United States of America

Xiuliang Liang

China

Jun Liu

China

Weiguo Liu

China

Bill Lei Lu

United States of America

Daqing Ma

United Kingdom

Jianfang Ma

China

Ken Marek

United States of America

Fei Miao

China

Jing Pan

China

Bruce R. Ransom

United States of America

Huifang Shang

China

Wei Shen

United States of America

Jeremy Stern

United Kingdom

Bomin Sun

China

EK Tan

Singapore

Yuyan Tan

China

Beisha Tang

China

Jinzhou Tian

China

Ning Wang

China

Kai Wang

China

Xiaochu Wang

China

Kang Wenyan

China

Dirk Woitalla

Germany

Chengbiao Wu

United States of America

Weiming Xia

United States of America

Xunde (Daniel) Xian

United States of America

Xianwen Chen

China

Mingming Xu

China

Zuoshang Xu

United States of America

Min Ye

China

Yong Zhang

United States of America

Yu Zhang

China

Jialin Zheng

United States of America

Jian Zhong

United States of America

Chunjiu Zhong

Christmas Island

Xiongwei Zhu

United States of America

